# Navigating the Complexity: Advancing Diagnostic Strategies for Avian Reovirus in Chinese Poultry

**DOI:** 10.3390/ani16040553

**Published:** 2026-02-11

**Authors:** Qingsen Wang, Lingyue Zheng, Guangju You, Hui Dong, Shaoying Chen, Shao Wang, Shilong Chen

**Affiliations:** 1 Institute of Animal Husbandry and Veterinary Medicine, Fujian Academy of Agriculture Sciences, Fuzhou 350000, China; 2 Fujian Animal Diseases Control Technology Development Center, Fuzhou 350000, China; 3 College of Animal Sciences, Fujian Agriculture and Forestry University, Fuzhou 350000, China

**Keywords:** avian reovirus, diagnosis, genetic diversity, Chinese poultry, co-infection, emerging technologies

## Abstract

Avian reovirus (ARV) infections pose a significant and evolving threat to China’s poultry industry, the world’s largest. Diverse farming systems—ranging from modern intensive operations to traditional waterfowl-poultry polyculture—foster a unique ecological niche for ARV, defined by complex serotypic and genotypic diversity, marked regional variations, potential interspecies transmission between chickens and waterfowl, and recurrent co-infections. Collectively, these factors undermine the efficacy of conventional diagnostic approaches. This review systematically outlines the current epidemic landscape of ARV in China, highlighting the molecular characteristics of prevailing strains (particularly those from waterfowl) and their roles in diagnostic evasion. We critically assess the performance and limitations of existing diagnostic techniques (virus isolation, ELISA, PCR/qPCR) within the Chinese epidemiological setting. Furthermore, we discuss innovative technologies—including multiplex qPCR, CRISPR-Cas systems, and next-generation sequencing (NGS)—that offer potential for developing next-generation diagnostics tailored to China’s specific challenges. Finally, we propose future directions, with an emphasis on standardization, data sharing, and interdisciplinary collaboration to bridge the gap between cutting-edge innovation and on-farm application for precise ARV control.

## Simple Summary

China’s poultry industry, the world’s largest, faces serious threats from avian reovirus infections. The virus thrives in diverse farming systems—from dense large-scale farms to small mixed flocks of chickens, ducks, and geese—leading to many virus types, cross-species spread, and frequent co-infections that make traditional detection methods slow or inaccurate. This review aims to summarize existing detection tools, highlight new technologies, and suggest future improvements. We found traditional methods have key limits, while innovative tools like rapid molecular tests and gene sequencing better address China’s unique challenges. To improve control, we need standardized detection protocols, shared virus data, and closer collaboration between researchers and farmers. These advances will help quickly identify infections, reduce economic losses for farmers, and support a stable, safe poultry supply—benefiting both the industry and the public.

## 1. Introduction

China is the world’s leading poultry producer. As of 2023, its poultry industry maintained steady growth: 7.195 billion white-feathered broilers were slaughtered, yielding ~4.673 million metric tons of meat; the laying hen inventory reached 1.189 billion, producing 28.39 million metric tons of eggs annually; 4.218 billion meat ducks and 515 million geese were slaughtered, with 149 million laying ducks retained [1]. This massive industry relies on a highly diverse range of farming systems, spanning large-scale intensive operations to smallholder mixed-species households, coupled with a tiered disease prevention and control program tailored to production scales. Large-scale farms (e.g., broiler complexes in Shandong and Hebei provinces) implement standardized biosecurity protocols, including all-in/all-out production cycles, routine disinfection of premises and equipment, serological monitoring of key pathogens, and mandatory vaccination against high-priority diseases (e.g., avian influenza, Newcastle disease). In contrast, smallholder farms—predominant in southern rural areas—often adopt low-cost, low-biosecurity practices, with irregular vaccination schedules, limited pathogen surveillance capacity, and frequent cross-species contact between chickens, ducks, and geese. This structural dichotomy directly amplifies the challenges of diagnosing Avian Reovirus (ARV) infections in China. On one hand, the high-density environment in large-scale broiler farms (30 birds/m^2^) drives a 63% co-infection rate between ARV and Fowl Adenovirus serotype 4 (FAdV-4) [2]; the superimposition of clinical signs (e.g., joint swelling + liver hemorrhage) complicates the differential diagnosis of individual pathogens. On the other hand, small-scale “chicken-duck-goose mixed-farming” in Fujian and Guangdong provinces facilitates ARV interspecies transmission. For example, in 2020, the novel duck reovirus (NDRV)-YF10 strain was isolated from Muscovy ducks in South China’s coastal regions; phylogenetic analysis based on the S1 gene revealed its close relatedness to chicken-origin ARV strains [3]. Furthermore, cross-regional transportation (averaging 1 billion poultry annually) has accelerated ARV genotype spread, with six genotypes co-circulating across broad geographic areas of China [4]. These China-specific challenges make “accurate, rapid, and multi-target ARV diagnostic technologies” a core breakthrough for epidemic prevention and control.

ARV poses a significant threat not only to China’s chicken industry but also to its rapidly expanding waterfowl sector—which contributes 61% of global duck meat and 87% of global goose meat production [5]. In chickens, ARV infections are associated with viral arthritis, tenosynovitis, runting-stunting syndrome (RSS), malabsorption syndrome, and respiratory/enteric diseases [6,7,8]. Since 2018, outbreaks of NDRV and goose-origin Muscovy duck reovirus (Go-MDRV) have caused annual losses exceeding 5 billion RMB in southern China, with mortality rates of 10–90% in 14–21-day-old ducklings and 30–50% in goslings [6,9,10,11]. Chicken-derived ARV and waterfowl-derived ARV (including NDRV, C-MDRV, Go-MDRV) exhibit low sequence homology, and vaccines developed based on chicken ARV strains (e.g., S1133) fail to induce protective immunity against waterfowl ARV infections. Diagnosing ARV in China presents unprecedented challenges distinct from global contexts, rooted in five key factors: serotypic/genotypic complexity [12,13,14], regional heterogeneity [4,15], interspecies transmission [16,17,18], prevalent co-infections [18], and vertical transmission [19].

ARV belongs to the genus Orthoreovirus of the family Reoviridae, with a non-enveloped icosahedral capsid (55–80 nm in diameter) enclosing a genome composed of 10 segments of double-stranded RNA (dsRNA). These segments are classified into three size classes: large (L1–L3), medium (M1–M3), and small (S1–S4), encoding 12 proteins including 8 structural proteins (λA-λC, μA-μB, σA-σC) and 4 non-structural proteins (μNS, σNS, p10, p17) [20]. ARV exhibits high genetic diversity in China. At least six ARV genotypes (I–VI) circulate in chickens [12,13], While waterfowl harbor distinct lineages (e.g., NDRV, Classical Muscovy duck reovirus [C-MDRV]) with low sequence homology to chicken strains [21,22]. For instance, the *σC* gene—a key genotyping marker—shows substantial sequence variation across ARV genotypes and lineages (Table 1). This genetic diversity hinders the development of universally effective diagnostic assays, as primers/probes designed for one genotype may fail to detect others [23].

China’s ARV epidemiology is complex and geographically variable. For example, multiple endemic subtypes coexist in regions like Shandong [4], while waterfowl-specific strains (MDRV/NDRV) dominate southern provinces (e.g., Guangdong, Fujian) where interspecies transmission is more frequent [27]. This regional heterogeneity necessitates region-specific diagnostic approaches, conflicting with the need for standardized testing protocols.

ARV interspecies transmission is a prominent issue in China. Chicken-origin ARV strains have been identified in Peking ducks—exemplified by NDRV-TH11, isolated from Peking ducks in southeastern China. This strains shares higher *σB* gene homology with chicken reference strains than with C-MDRV [28]. Such transmission complicates diagnostic specificity: assays developed for chicken strains may fail to detect waterfowl-derived variants infecting chickens, and vice versa.

ARV frequently co-infects poultry with other pathogens in China. In chickens, co-infects with fowl adenovirus serotype 4 (FAdV-4), infectious bursal disease virus (IBDV) and Staphylococcus aureus are common [2,29,30]. In waterfowl, co-infections with Salmonella Typhimurium, goose astrovirus (GAstV), FAdV, H9N2 subtype avian influenza virus (AIV), and goose parvovirus (GPV) are common [31,32]. These co-infections mask or mimic ARV-specific clinical signs and pathological changes, leading to diagnostic delays or misidentification.

This comprehensive review examines how China’s unique epidemiological landscape shapes ARV diagnostic challenges and evaluates innovative technologies developed to address these complexities. By systematically linking China’s poultry production characteristics to diagnostic hurdles, we aim to provide a foundation for advancing ARV detection methods in one of the world’s most important poultry-producing regions.

## 2. Epidemiological Landscape of ARV in China: Targets for Diagnosis

ARV is widely distributed in chicken flocks across multiple regions of China. Between 2010 and 2013, Yu et al. conducted ARV antibody detection viaELISA on chicken serum samples from 19 provinces/municipalities nationwide. Positivity rates exceeded 90% (15,860/17,058) in all regions except Shandong and Guangdong, confirming the widespread prevalence of ARV in Chinese chicken populations [33]. Surveillance in 16 provinces in China from 2019 to 2020 showed that genotype I of ARV had the highest prevalence, and the main infection host of these positive samples was white-feathered broilers. No ARV strains were detected in layer hens and yellow-feathered broilers. The onset time is mainly at 15–45 days of age. The main affected provinces are located in eastern China, including Shandong, Jiangsu, Anhui, Fujian, Hebei, Henan, Heilongjiang, Jilin and Liaoning [4].

Beyond chickens, ARV infects other avian species—most notably ducks. In 2016, a reovirus was isolated from a duck farm in Shandong Province. This virus replicated efficiently in duck embryos and duck embryo fibroblast (DEF) cells, with syncytium formation observed. Genomic analysis revealed a canonical structural organization but distinct genetic divergence from previously reported Chinese duck-origin reoviruses. Phylogenetic analysis clustered it most closely with the German duck reovirus strain D253/6/1-10, forming a monophyletic clade separate from known avian reovirus references [34]. Detection of 1970 goose samples from 9 provinces/municipalities directly under the Central Government from 2018 to 2021 found that the prevalence rate of Go-MDRV was 18.22% (359/1970), and the prevalence rate of NDRV in Guangdong and Fujian reached 29.5% [32]. These findings highlight ARV’s broad host range and genetic heterogeneity across avian species—key considerations for diagnostic assay design.

Relevant surveillance and isolation studies have been conducted across diverse Chinese provinces/municipalities Zhang et al. isolated 18 emerging ARV variants from tendons and joint synovial fluid of broilers with arthritis/tenosynovitis from commercial farms. *σC* gene sequence analysis showed 4 isolates clustered with the vaccine strain S1133 (Cluster 1), while 14 grouped into Clusters 2, 3, and 6. Field isolates shared notably low sequence identity (38.1–81.9%) with S1133, with Cluster 6 isolates exhibiting the lowest homology (38.1–67.2%). Pathogenicity testing in specific pathogen-free (SPF) chickens demonstrated that these variants—especially Cluster 6 strains—were more virulent and induced higher incidence rates than S1133 [13]. This indicates rapid ARV evolution and increasing virulence in China, with emerging variants posing greater threats to the poultry industry.

In summary, ARV exhibits wide distribution and high infection rates across China’s poultry industry. Infection dynamics vary by poultry species, with the virus showing clear trends of genetic variation and enhanced pathogenicity [35,36,37]. While China’s ARV epidemiological landscape is defined by unique complexities, brief comparisons with other major poultry-producing regions highlight both shared and distinct challenges: Southeast Asia, with similar mixed farming systems, faces significant co-infections of ARV with AIV and Newcastle disease virus, yet exhibits relatively narrow genotypic diversity and low prevalence of waterfowl-specific variants. Europe’s intensive monogastric poultry farming mitigates interspecies transmission risks, with ARV monitoring centered on broiler tenosynovitis and supported by standardized EU-wide diagnostic protocols. North America features high ARV genetic diversity but limited waterfowl ARV circulation. These epidemiological characteristics—namely a broad host range, marked genetic heterogeneity, and evolving virulence—have direct implications for the selection of reliable diagnostic targets and pose significant challenges to the sustainable and healthy development of the poultry industry. This underscores the need for enhanced ARV surveillance, further research, and optimized prevention/control strategies tailored to China’s unique epidemic landscape.

## 3. Existing Diagnostic Methods in China: Performance and Limitations

### 3.1. Virus Isolation: The Gold Standard with Practical Constraints

Low viral loads in clinical samples often hinder ARV isolation. For example, during late-stage infections or mild cases, the virus exhibits uneven tissue distribution and limited abundance—further complicating isolation efforts. Notably, vertical transmission-induced infections in neonates exacerbate this limitation: ARV transmitted via eggs maintains extremely low titers in embryonic tissues (e.g., liver, spleen, and yolk sac) and exhibits focal distribution, making it difficult to obtain sufficient viral particles for in vitro cultivation even with optimized sample processing. Additionally, vertically transmitted ARV may enter a latent state in neonatal tissues, further reducing the efficiency of virus isolation. When isolating the ARV-SD26 strain, extensive sample processing and screening were required to achieve successful isolation, highlighting that low viral loads pose a practical barrier to ARV recovery [38]. Beyond low viral loads, several other critical factors constrain the success of ARV isolation, even in samples with high viral titers. First, inhibitors present in clinical samples—such as proteases in tissue homogenates and polysaccharides in fecal specimens—can directly inhibit viral replication in vitro, compromising isolation efficiency. Notably, high viral load alone does not guarantee successful isolation. Even when the viral titer reaches 10^5^ copies/mL, samples containing high concentrations of fecal inhibitors show an isolation rate of less than 60%, resulting in false-negative outcomes. ARV displays distinct host cell tropism, with not all cell lines supporting its growth. Commonly employed cell lines include chicken embryo liver (CEL) cells and the chicken hepatocellular carcinoma (LMH) cell line; however, growth characteristics of ARV strains vary across these substrates. Zhao et al. noted that ARV strains of different genotypes exhibit distinct replication kinetics in LMH cells [39]. This necessitates selecting cell lines tailored to the target strain’s properties, adding complexity to viral cultivation. Some ARV strains may grow slowly or fail to propagate in specific cell lines, resulting in ineffective isolation and culture [39].

When ARV is cultured in vitro, several days typically elapse from sample inoculation to the observation of distinct cytopathic effect (CPE). For example, chicken embryo culture requires 3–7 days to detect embryonic pathological changes [40]. This lengthy turnaround time impedes timely disease diagnosis and may delay critical prevention and control interventions—aligning with the urgent need for rapid diagnostics in transboundary disease management. To obtain sufficient viral quantities for subsequent identification and research, multiple passages are often required. Each passage demands a specific culture period, further extending the overall isolation and culture cycle. For instance, 3–5 passages (with 1–2 days between each passage) are commonly needed for ARV purification and propagation, prolonging the entire process to several weeks [41].

Observation of CPE alone is insufficient to definitively confirm the identity of ARV following isolation. Instead, definitive confirmation requires an integrated approach combining molecular techniques—such as reverse transcription–polymerase chain reaction (RT-PCR) and nucleic acid sequencing—with serological verification, including virus neutralization test tests. Tsutsumi et al. emphasized that viral isolation must be complemented by additional assays for definitive identification, a requirement that also applies to ARV [42]. This multiplies the complexity and workload of the process, as each technique necessitates specialized personnel and dedicated equipment.

To address species-specific isolation differences: Chicken ARV exhibits optimal growth in CEL and LMH cell lines, whereas waterfowl ARV (NDRV/Go-MDRV) requires DEF cells or QT-35 cell line for reliable isolation. Corresponding to cell line preferences, chicken ARV induces distinct CPE in chicken embryos within 3–5 days, while waterfowl ARV requires an extended incubation period of 5–7 days to show detectable embryonic lesions. These differences arise from species-specific viral tropism, necessitating tailored isolation protocols for gallinaceous birds vs. waterfowl.

### 3.2. Serological Assays: ELISA as the Workhorse, but with Specificity Gaps

The rising prevalence of ARV variants and their enhanced pathogenicity have exposed critical limitations in current ARV control strategies [43]. Notably, all commercial enzyme-linked immunosorbent assay (ELISA) platforms are developed using classical ARV strains as targets, rendering them unable to reliably detect variant strains. Currently, all commercialized ELISA detection reagents are developed using antigens from classic chicken-derived virus strains, but these methods cannot detect waterfowl ARV antibodies. Therefore, there is an urgent need to develop detection kits targeting specific antigens of waterfowl-derived avian reovirus (such as the σC protein of NDRV) [44]. This deficiency severely compromises accurate diagnosis, timely intervention, and effective containment during variant ARV outbreaks. Compounding this issue, the classical ARV vaccine strain S1133—currently the gold standard for ARV immunization—fails to confer protective immunity against variant strain challenges [45,46]. As a result, immunized flocks remain susceptible to clinical disease, further undermining ARV management efforts. Given that existing classical strain-targeted ELISAs cannot meet the detection needs of variant ARVs, there is an urgent demand for specialized ELISA-based antibody assays tailored to these emerging variants [47].

A key limitation of conventional ELISAs lies in their failure to discriminate between antibodies induced by wild-type ARV infection and those generated via vaccination (e.g., the widely used S1133 strain), a gap that is exacerbated by the widespread implementation of vaccination programs in Chinese poultry flocks. A further complication arises from vertical transmission: breeder flocks infected with ARV transmit maternal antibodies to neonates via egg yolk, which interferes with serological detection in young birds (≤2 weeks old). Conventional ELISAs cannot distinguish maternal antibodies derived from vertically transmitted ARV from antibodies induced by post-hatch active infection, leading to false-positive results for infection status or misclassification of latent vertical infection as passive antibody acquisition. This ambiguity is particularly problematic for early epidemic warning in commercial broiler and waterfowl flocks. This ambiguity directly hinders accurate epidemic assessment: positive ELISA results in immunized populations cannot confirm the circulation of active wild-type strains, leading to misjudgments of epidemic dynamics and impeding the formulation of targeted prevention and control strategies. The issue is particularly pronounced in waterfowl: vaccines developed based on chicken-derived ARV strains offer no protective immunity against waterfowl-specific variants (NDRV/Go-MDRV), yet cross-reactive antibodies induced by chicken-vaccine exposure can trigger false-positive signals in waterfowl serological tests, further confounding diagnostic accuracy. Multiple studies on vaccine efficacy and disease surveillance have corroborated this shortcoming, noting that conventional ELISAs cannot robustly differentiate infected from immunized animals—necessitating integration with supplementary detection methods for comprehensive judgment [48]. Given the critical role of such differentiation in precise ARV control, this remains a major unresolved deficiency of current ELISA-based diagnostic approaches.

Beyond ELISA, other serological techniques—including Agar Gel Immunodiffusion (AGP) and Indirect Immunofluorescence Assay (IFA)—have been applied for ARV antibody or antigen detection. The AGP test, based on antigen–antibody precipitation in a gel matrix, is simple to perform and requires no specialized equipment. However, its primary drawback is low sensitivity: it may miss infections with low antibody titers or those in early stages, leading to false-negative results [49]. In contrast, IFA offers higher sensitivity than AGP and enables visual localization of viral antigens in infected cells. Nevertheless, IFA is prone to non-specific fluorescence (increasing false-positive risks), requires expertise in pattern recognition, and relies on fluorescence microscopes. Additionally, the subjective nature of result interpretation limits its applicability in routine or resource-constrained field settings—particularly relevant for cross-regional poultry disease surveillance [50].

### 3.3. Molecular Assays: qPCR as the Mainstay, but Target-Dependent Limitations qPCR

qPCR is the mainstay of molecular detection for ARV, offering high sensitivity and rapid turnaround compared to traditional methods. However, its performance is inherently dependent on the target gene selected—a critical limitation exacerbated by ARV’s high propensity for genetic recombination and reassortment during natural infections [51,52,53]. If the qPCR target gene region undergoes recombination or reassortment, primers and probes may fail to bind specifically to the modified gene sequence, leading to false-negative results. For vertically transmitted ARV, the virus tends to distribute unevenly in embryonic or neonatal tissues (e.g., scattered foci in lung, kidney, or intestinal tissues) and maintains low replication levels (often <10^2^ copies/μL in neonatal samples), which increases the risk of false-negative results in qPCR. The focal distribution of the virus also means that random sampling of a single tissue (e.g., liver alone) may miss the infected foci, further compromising the reliability of molecular detection for vertical transmission events. From a viral genetics perspective, frequent genetic reassortment (a hallmark of ARV evolution) challenges detection methods relying on conserved traditional target genes, directly compromising detection sensitivity and specificity [54]. As research into ARV genetic variation and recombination advances, this target-dependent limitation is likely to become increasingly prominent—particularly amid the cross-species transmission and regional spread of ARV variants in China.

### 3.4. Pathological Diagnosis: Basic Tools for Preliminary Screening

In grassroots poultry farms and field surveillance scenarios, pathological diagnosis serves as the most direct, simple, and widely adopted preliminary screening method for ARV, effectively complementing the limitations of virus isolation (complex operation, long turnaround time) and molecular/serological assays (dependency on equipment and specialized reagents). Its core value lies in providing rapid etiological clues through visual observation of characteristic lesions, which is particularly critical for resource-constrained rural areas in China where advanced diagnostic tools are often inaccessible. Notably, ARV infections in both gallinaceous birds and waterfowl share similar post-mortem findings in key organs (spleen, liver, tendons, etc.), necessitating careful differentiation to avoid misdiagnosis. For gallinaceous birds (predominantly chickens), typical lesions include: symmetric swelling of hock and toe joints, hemorrhage and edema of the tendon sheath (especially in broilers with tenosynovitis); splenomegaly with white necrotic foci; multifocal necrotic lesions in the liver; and in cases complicated by runting-stunting syndrome (RSS), stunting and intestinal mucosal thinning [55]. For waterfowl (ducks and geese), lesions are predominantly distributed in visceral organs with overlapping features: multiple grayish-white focal necroses in the liver and spleen, myocardial hemorrhage and degeneration, hyperemia of the intestinal mucosa [56,57]; additionally, goslings infected with Go-MDRV may show kidney swelling and urate deposition, a feature less common in chickens. Microscopic examination further confirms ARV-associated tissue damage: in chicken joint tissues, there is synovial cell hyperplasia, fibrin exudation, and extensive lymphocyte infiltration in the tendon sheath [58,59]; in waterfowl visceral organs, pathological changes include coagulative necrosis of hepatocytes with surrounding eosinophil and monocyte infiltration, myocardial fiber fragmentation and interstitial inflammation, and intestinal mucosal epithelial cell shedding [11]. As a preliminary screening tool, pathological diagnosis relies on characteristic lesions to narrow down potential pathogens but cannot distinguish ARV from other pathogens with similar lesions (e.g., FAdV-4-induced liver hemorrhage, GPV-induced goose visceral necrosis). Therefore, positive pathological findings must be confirmed by serological assays (e.g., IFA) or molecular methods (e.g., RT-qPCR) to achieve definitive diagnosis, forming a complementary diagnostic chain with the aforementioned techniques to improve diagnostic accuracy in complex field environments.

## 4. Innovative Diagnostic Technologies: Addressing Chinese Challenges

### 4.1. Multiplex Digital qPCR/Microfluidic Chips: Tackling Co-Infections and Genotype Diversity

Co-infections represent one of the most pressing diagnostic challenges in Chinese poultry farms—driven by high-density production systems and mixed farming models. This context positions multiplex digital PCR (dPCR)—which detects multiple targets in a single reaction—as a critical innovation tailored to China’s unique epidemiological needs. Li et al. developed a multiplex dPCR assay targeting the C gene of Duck Tembusu virus (DTMUV), the rep gene of duck circovirus (DuCV), and the S3 gene of novel duck reovirus (NDRV). Sensitivity testing revealed a limit of detection (LOD) of 1.3 copies/μL, a 10-fold improvement over multiplex qPCR. Field validation with 173 clinical samples showed positive detection rates of 18.5% (32/173) (DTMUV), 29.5% (51/173) (DuCV), and 14.5% (25/173) (NDRV)—approximately 4% higher than those from multiplex qPCR [60]. This improvement is of great value for the monitoring of mixed infections, which are common in high-density aquaculture systems in China. But dPCR field applicability remains constrained by high resource requirements—specialized equipment and trained personnel are largely limited to provincial reference laboratories, and it lacks formal veterinary regulatory approval in China, hindering widespread adoption beyond research and surveillance contexts.

Despite these advantages, dPCR faces notable barriers to widespread veterinary application in China. First, high costs place a significant economic burden on animal health institutions: dPCR instruments are 1.5–2 times more expensive than qPCR platforms, with dedicated reagents and consumables also commanding premium prices. Second, throughput limitations hinder scalability—most dPCR systems process ≤ 8 samples per run, failing to meet high-throughput screening demands during large-scale epidemics, which are common in China’s geographically extensive poultry industry. Third, operational complexity presents a bottleneck: precise droplet generation and amplification require specialized technical proficiency, and the process is more time-consuming than qPCR, limiting adaptability for rapid on-farm testing. Fourth, low standardization complicates result consistency: the veterinary field lacks unified operating protocols for dPCR and consensus on diagnostic thresholds, leading to interpretation variability across platforms. Finally, poor sample adaptability undermines reliability—while dPCR tolerates inhibitors better than qPCR (Table 2), fecal polysaccharides and tissue homogenate proteases still impair droplet generation efficiency, requiring optimized pretreatment to avoid false-negative outcomes.

A tripleplex microfluidic chip targeting three highly pathogenic swine diseases (caused by Porcine Reproductive and Respiratory Syndrome Virus (PRRSV), Classical Swine Fever Virus (CSFV), and Porcine Circovirus Type 2 (PCV2)) has been developed and applied. A single chip can process 4 different samples simultaneously, complete the full-process detection in 12 min, and require only 4 μL of sample volume per sample; additionally, it can achieve an accuracy of >93.8%, a sensitivity of >88.8%, and a specificity of >96.6% [61]. Although microfluidic chips for the detection of avian diseases (including those caused by ARV) have not yet been developed, the application of this technology in the swine industry provides an important reference for the rapid diagnosis of large-scale waterfowl diseases in China. However, the implementation of these technologies in China still faces challenges. First, there is a demand for dedicated equipment: microfluidic chips require supporting readout devices (costing approximately 200,000 RMB), which limits their popularization in laboratories with limited resources. Second, technical expertise is required: the operation and result interpretation of these technologies demand a higher level of skills compared with traditional methods. Third, there is a need for customization: detection targets must be updated regularly to cope with the evolution of pathogen populations. Despite these challenges, multiplex qPCR and microfluidic chips represent significant advancements for addressing China’s high co-infection rates and diverse pathogen landscape. Their ability to simultaneously detect multiple pathogens aligns well with the complex diagnostic needs of Chinese poultry farms.

### 4.2. CRISPR-Cas Technologies: Flexibility for Rapidly Evolving ARV Strains and Transboundary Surveillance

CRISPR-Cas-based diagnostics have emerged as a game-changing tool for detecting rapidly evolving pathogens—addressing a core challenge of ARV control in China, where frequent cross-species transmission and genetic recombination drive the emergence of novel variants with transboundary spread potential. In 2024, Zhang et al. developed a dual-target detection assay combining Recombinase Polymerase Amplification (RPA) with CRISPR-Cas12a/Cas13a, targeting the VP1 gene of Duck Hepatitis A Virus Type 3 (DHAV-3) and the S3 gene of NDRV—a key ARV lineage threatening China’s waterfowl industry [62]. This single-tube integrated system eliminates aerosol contamination risks—a critical advantage for field-based surveillance—and achieves a limit of detection (LOD) as low as 100 copies/μL for both pathogens. Notably, the assay shortens total detection time from 80 min to 35 min, outperforming conventional qPCR in turnaround time. Validation with 24 clinical samples confirmed 100% consistency with qPCR results, underscoring its reliability. The assay’s high specificity, sensitivity, and user-friendliness make it uniquely suited for on-site detection in China’s diverse poultry farming systems—from intensive operations to small-scale mixed farms. These attributes are particularly valuable amid high-density production and frequent cross-species transmission, which accelerate ARV evolution and create urgent needs for point-of-care diagnostics that can keep pace with emerging variants. For transboundary disease control, such rapid, field-deployable tools enable timely identification of ARV outbreaks at source, mitigating the risk of cross-regional or international spread.

A CRISPR-Cas13a-based point-of-care testing (POCT) assay—integrated with lateral flow chromatography—has been developed for novel duck reovirus (NDRV) detection, addressing critical gaps in China’s rural and resource-limited poultry farming settings [63]. Its consistency with qPCR reached 100%. Meanwhile, it showed no cross-reactivity with Duck Hepatitis A Virus (DHAV), DTMUV, and novel duck-origin Goose Parvovirus (nD-GPV). This method does not require specialized equipment and can yield results within 40 min—a characteristic that makes it suitable for remote areas with limited laboratory resources, which is in line with the practical needs of the extensive rural poultry farming industry in China.

The CRISPR-Cas technology faces practical constraints in poultry farm settings, including reagent storage requirements, qualitative-only detection, and sample pretreatment challenges [64,65,66].

### 4.3. Next-Generation Sequencing (NGS): Uncovering Unknown Pathogens and Tracing Transmission

Next-generation sequencing (NGS) has revolutionized ARV diagnostics and epidemiological surveillance in China by providing capabilities beyond those of conventional methods. These include the detection of novel viral variants and high-resolution tracing of transmission chains, both of which are essential for effective transboundary disease control. By capturing full genomic data, NGS addresses core challenges of China’s complex ARV landscape: rapid viral evolution, cross-species spillover, and cross-regional spread. Notably, during 2020 broiler arthritis outbreaks in Anhui and Shandong Provinces, initial RT-PCR confirmed ARV positivity but ruled out common avian pathogens (AIV), Newcastle Disease Virus (NDV), Avian Adenovirus (AAV), and Infectious Bronchitis Virus (IBV)). NGS analysis subsequently identified two distinct ARV variants (designated 0543 and SDYT), with only 55.4% nucleotide homology in the *σC* gene—classifying them into genotype I and genotype IV, respectively [53]. After conducting viral isolation and identification on two dead black swans from Chengdu Zoo, ARV infection in black swans was discovered for the first time. Subsequent NGS revealed that the *σC* gene of this ARV shared a homology of up to 98.5% with NDRV strains that have emerged recently. This genomic evidence confirmed the cross-species transmission event between black swans and ducks, which provided guidance for the formulation of targeted biosecurity measures. Such in-depth insights hold significant value for monitoring cross-species transmission between poultry farming and wild birds in China [16].

In the context of China’s complex ARV epidemiology—characterized by high co-infection rates, frequent genetic recombination, and cross-regional/cross-species transmission—NGS offers three distinct values that align with transboundary disease control priorities. First, it enables rapid identification of etiological agents during atypical disease outbreaks, critical for early response to potential transboundary epidemics. Second, it supports precise characterization of emerging ARV variants, facilitating the timely detection of novel strains that may evade conventional diagnostics and spread across borders. Third, it maps cross-regional and cross-species transmission chains, providing actionable insights for targeted biosecurity and precision prevention—addressing a key challenge in mitigating transboundary ARV risks. NGS’s utility in China stems from three core advantages tailored to the country’s diversified poultry production systems. First, unbiased detection eliminates the need for prior target knowledge, enabling simultaneous screening of all potential pathogens—ideal for China’s high co-infection environments and the detection of unknown or emerging ARV recombinants. Second, whole-genome analysis delivers comprehensive genetic data, resolving evolutionary relationships and transmission dynamics that underpin cross-species spillover and transboundary spread. Third, multipathogen detection capability directly addresses the prevalent co-infections in Chinese poultry farms, avoiding misdiagnosis that could hinder effective transboundary disease control. Collectively, these advantages make NGS indispensable for mitigating risks posed by ARV recombination and cross-species transmission—key drivers of transboundary disease emergence.

Despite its transformative potential, NGS faces three pragmatic barriers to widespread application in China’s ARV surveillance and transboundary control efforts. First, high costs (≈1500 RMB per sample) limit its use as a routine monitoring tool, particularly for resource-constrained regional laboratories and rural areas—frontline nodes for transboundary disease detection. Second, prolonged turnaround times (3–5 days for results) hinder emergency response to acute outbreaks, where rapid intervention is critical to curbing cross-regional spread. Third, data analysis requires specialized bioinformatics expertise and supporting infrastructure, which are lacking in many underdeveloped regions—creating disparities in transboundary surveillance capacity across China’s geographically extensive poultry industry.

### 4.4. Big Data and AI: Predicting Transboundary Trends and Optimizing ARV Diagnostics

China’s expansive poultry industry generates enormous volumes of ARV -related data—including genomic sequences, epidemiological records, and diagnostic results. Artificial Intelligence (AI) and big data analytics are increasingly harnessed to translate this data for improved diagnostics and outbreak prediction. Maryam et al. developed a machine learning model for reovirus type classification [67], using 2290 sequences of Turkish ARV strains as training data. This model exhibited excellent classification performance, with an average accuracy of 92%, an F1-Macro (F1-score for macro-averaging) of 93%, and an F1-Weighted (F1-score for weighted-averaging) of 92%. Meanwhile, in the analysis of σC, the random forest algorithm with k-mer encoding achieved the optimal results, with corresponding accuracies of 0.92%, 0.93%, and 0.92% respectively. This research outcome holds significant reference value for China’s diagnostic system in addressing the rapid evolution of ARV strains.

Beyond genotype classification, AI and big data offer unique value for transboundary ARV control in China. They can integrate multi-source data (e.g., cross-regional flock movement records, climatic factors, and genomic data) to predict outbreak trends, identifying high-risk areas for transboundary spread before clinical signs emerge. Additionally, AI-driven analytics can optimize diagnostic assay design by identifying conserved genetic regions across diverse ARV strains—addressing the target-dependent limitations of conventional methods and enhancing detection coverage for cross-species or recombinant variants. These capabilities align directly with China’s need for precision diagnostics and proactive transboundary risk mitigation in its geographically extensive and ecologically diverse poultry production systems. The on-site applicability of AI-ML genotyping models is moderate. This is because cloud-based analysis can alleviate hardware limitations, but the regulatory guidelines for artificial intelligence-driven diagnostic tools in veterinary medicine are still in the early stages of development, and there is no clear approval pathway in China.

## 5. Future Directions for ARV Diagnostics in China

The future development of ARV diagnostics in China will focus on four core and feasible directions, advancing in phases with practical adaptation as the key principle. In standardization construction, the current lack of localized standards must be addressed by prioritizing high-priority tasks: developing national reference strains for 3–5 major prevalent ARV genotypes (covering key chicken and waterfowl strains) and matched positive controls (viral nucleic acids, antisera) to replace incompatible international strains like S1133. Simultaneously, simplified national diagnostic protocols should be issued, clarifying sample collection specifications (liver/spleen for waterfowl, joint fluid for chickens), unified target genes (μA for universal detection, σC for genotyping), and basic quality control requirements. Supporting systems such as sample storage/transport guidelines and inter-laboratory proficiency testing will be improved gradually, focusing first on applicability in grassroots laboratories rather than pursuing full coverage immediately.

At the resource sharing level, low-cost and high-practicality measures are needed to break data silos. A national ARV core database will be established, initially integrating whole-genome sequences of prevalent strains, key epidemiological data (region, host, clinical signs), and diagnostic performance of mainstream methods to avoid overloading with redundant information. Regional biological resource banks will be set up in major poultry-producing provinces to share representative live viruses, inactivated antigens, and typical clinical samples, reducing redundant isolation work. Regular data exchange among provincial veterinary laboratories will be promoted through a centralized online portal, with international cooperation limited to core technology validation to avoid overambitious global applicability goals.

To bridge the gap between laboratory innovation and on-site application, interdisciplinary collaboration must focus on solving “last-mile” problems. A regular communication mechanism will be established between diagnostic developers and end-users (veterinarians, farmers) to optimize technologies for on-site conditions—for example, simplifying CRISPR-based POCT sample pretreatment to adapt to mixed farming environments with limited equipment. Regional diagnostic demonstration centers will be set up in 5–8 major poultry provinces, undertaking technology promotion, on-site training, and feedback collection, without attempting nationwide coverage due to resource constraints. Targeted farmer training programs will be launched, focusing on early symptom recognition and standardized sample collection aligned with national protocols, rather than complex diagnostic principles.

Precision diagnostics will advance in phases, abandoning overambitious “comprehensive characterization” goals for incremental progress. In the short term (1–3 years), priority will be given to developing reliable vaccine-wild strain differentiation technologies, such as SNP-qPCR targeting S1133 vaccine-specific mutations (low cost and highly compatible with existing qPCR platforms) and modified CRISPR-Cas13a POCT for small farms that require no specialized equipment. In the medium term (3–5 years), simplified pathogen characterization methods will be developed to simultaneously identify genotypes and key antigenic markers, avoiding premature integration of virulence assessment due to technical complexity. A basic diagnostic reporting system will be built, including infection status, genotype, and preliminary co-infection hints (instead of multi-parameter complex recommendations), with AI applications limited to auxiliary genotype classification using mature algorithms like random forest with k-mer encoding to reduce technical barriers.

Meanwhile, efforts to enhance the comprehensive diagnostic and monitoring early warning system will be strengthened, constructing a multi-level framework adapted to China’s farming reality. A workflow of “rapid screening → laboratory confirmation → key strain genotyping” will be adopted, using low-cost rapid kits (e.g., modified ELISA, simple CRISPR strips) for primary screening and reserving qPCR/NGS for confirmation and genotyping to balance efficiency and cost. A basic IoT-based remote diagnosis tool will be developed for grassroots farms, focusing on real-time collection of environmental data (temperature, humidity) and key health indicators without overcomplicating with full-range sensor deployment, enabling remote consultation with regional centers. A national ARV monitoring network covering major production areas will be constructed, conducting quarterly virus isolation and semi-annual sequencing to track mutation trends, and updating diagnostic reagents annually instead of real-time adjustments due to operational constraints. Finally, a simplified early warning model will be built by integrating core data (farming density, regional trade volume, historical epidemic records) to identify high-risk areas, avoiding over-reliance on complex meteorological or multi-source data due to data availability limitations.

## 6. Conclusions

China’s unique poultry farming landscape—characterized by diverse poultry species, high production density, and regional heterogeneity—poses distinct challenges for ARV diagnostics. Conventional methods (ELISA, single-plex qPCR) are becoming increasingly insufficient to detect China’s diverse and rapidly evolving ARV strains, as well as the frequent co-infections prevalent in its farming systems. However, innovative technologies—including multiplex qPCR, CRISPR-Cas, NGS, and AI—are being tailored to Chinese needs, providing targeted solutions to these pressing hurdles.

The way forward demands not only technical innovation but also systemic reforms: standardization of diagnostic reference materials, cross-regional data sharing, and enhanced collaboration between researchers and end-users. By aligning diagnostic developments with China’s unique epidemiological landscape, we can transition from “reactive detection” to “proactive precision diagnostics”—a pivotal step toward safeguarding China’s poultry industry and upholding global food security.

As China remains a global leader in poultry production, its strategy for ARV diagnostics is poised to serve as a model for other countries grappling with similar challenges. By sustaining technological innovation and interdisciplinary collaboration, China can develop diagnostic tools that effectively tackle its complex ARV epidemiological landscape—while advancing global efforts to mitigate the impact of this significant poultry pathogen.

## Figures and Tables

**Table 1:** Homology of *σC* genes among different ARV genotypes and lineages.

Genotype/Lineage	Host	*σC* Gene Characteristics
Genotype II	Chicken	70.9–76.0% homology with vaccine strain S1133 [12]
Genotype V		53.0–55.2% homology with vaccine strain S1133 [12]
Genotype IV		45.4–52.6% homology with vaccine strain S1133 [24]
NDRV	Duck (Muscovy)	Most amino acid mutations are located in the head domain of the σC protein, with a small number in the shaft domain [25]
Go-NDRV-GD2020	Goose	97.6% homology with C-MDRV [26]

**Table 2:** Performance Comparison of dPCR and qPCR for ARV Detection.

Aspect	qPCR	dPCR
Detection Sensitivity	High (capable of detecting copy-level targets)	Extremely high (capable of single-copy detection)
Absolute Quantification	Relies on standard curves for relative quantification	Does not require standard curves; enables absolute quantification
Inhibitor Tolerance	Susceptible to inhibitors (may cause Ct delay)	Higher tolerance to inhibitors
Throughput	High (96/384-well plates, high automation compatibility)	Low (limited parallel sample processing on most platforms)
Cost	Lower instrument and reagent costs	High initial investment and per-sample cost
Operational Simplicity	Standardized protocols, easily adaptable	Complex workflow requiring specialized training
Clinical Adoption	Widely adopted in veterinary diagnostics	Primarily limited to research; clinical validation remains insufficient

## Data Availability

No new data were created or analyzed in this study.
